# The Demands of Simple and Complex Arithmetic Word Problems on Language and Cognitive Resources

**DOI:** 10.3389/fpsyg.2021.727761

**Published:** 2021-10-18

**Authors:** Marian Hickendorff

**Affiliations:** Educational Sciences, Institute of Education and Child Studies, Leiden University, Leiden, Netherlands

**Keywords:** word problems, reading comprehension, arithmetic, mathematics, cognitive abilities, non-verbal reasoning, working memory

## Abstract

Solving arithmetic word problems requires constructing a situation model based on the problem text and translating that into a mathematical model. As such, word problem solving makes demands on students’ language comprehension and their domain-general cognitive resources. These demands may decrease when students get more experienced and use strategies that do not require fully understanding the situation presented in the problem. The current study aims to address this hypothesis. Students (*N*=444) from third to sixth grade solved a paper-and-pencil task with 48 mathematics problems, comprising symbolic arithmetic problems and standard word problems, as well as more complex word problems that involve two arithmetic steps or include irrelevant numerical information. Their performance was analyzed with multilevel logistic regression analyses. Results showed that within each grade, performance on the different problem types did not differ, suggesting that already in third-grade students seem helped nor hindered by presenting arithmetic problems in a story, even if that story contains irrelevant numerical information. Non-verbal reasoning was more important in standard word problems than in arithmetic problems in symbolic format in one-step arithmetic, and reading comprehension was more important in solving two-step arithmetic word problems than in one-step arithmetic word problems.

## Introduction

In contemporary mathematics education, arithmetic word problems (also called verbal or story problems) are omnipresent in instruction and assessment. Solving word problems is a complex, multi-phase process involving an interplay of various cognitive processes (Verschaffel et al., 2000, 2020). Central phases are the construction of a mental representation of the problem situation and the transformation of this situation model to a mathematical model, often a specific arithmetic expression (Kintsch and Greeno, 1985; Cummins et al., 1988; Verschaffel et al., 2000). These processes make demands on language abilities as well as domain-general cognitive resources (Fuchs et al., 2015, 2020; Wang et al., 2016). However, results in more experienced word problem solvers suggest that the steps of constructing a situation and mathematical model become less important, possibly because students use a more superficial strategy, relying heavily on their schemata for solving typical, one-step word problems that does not require fully understanding the situation (Hickendorff, 2013a). The current study aims to address this hypothesis by extending previous studies in three ways: by including students from a wider age range (third to sixth grade), by including more complex word problems (two-step arithmetic problems and problems including irrelevant numerical information), and by including a set of individual differences measures that taps into language comprehension and domain-general cognitive resources.

### Word Problems

Word problems in mathematics education are typically defined as verbal descriptions of a problem situation in which one or more questions are raised that can be answered by the application of mathematical operations that have been learnt at school on the numerical data that are available in the problem situation (Verschaffel et al., 2000, 2020). An example is “there are 136 persons at the party. To play a game they are distributed in groups of four persons. How many groups are formed?” Word problems play an important role in mathematics education for several reasons: They offer practice in applied problem solving and mathematical modeling in real-life situations, they can motivate students for mathematics, they train students to think creatively and develop their problem-solving abilities, and they can aid in the development of new mathematical concepts and skills (Verschaffel et al., 2000, 2020). However, word problems are also among the most difficult problems that students encounter. It is therefore not surprising that a large body of research has been devoted to word problems (for a recent review, see Verschaffel et al., 2020).

One of the branches of research focuses on the complex interplay of cognitive processes that play a role. Word problem solving models typically assume that the most critical steps in solving word problems are the construction of a mental representation of the problem situation (the situation model) and the translation of that situation model into a mathematical model (Kintsch and Greeno, 1985; Verschaffel et al., 2000). Leiss et al. (2019) provided empirical support for this claim by showing that constructing a situation model is crucial for the correct solution of word problems and takes a considerable amount of solution time, depending on the linguistic complexity of the tasks.

However, Hickendorff (2013a) found that students at the end of primary school did not show additional difficulties in solving word problems compared to solving their symbolically presented counterparts, nor did they use different strategies to solve the problems, nor did the problems have differential relations with reading comprehension. This suggests that students at the end of primary school did not perceive real differences between word problems and their symbolic counterparts. Hickendorff (2013a) attempted to reconcile the discrepancy between these patterns and the findings in younger students by the tentative explanation that the interplay between the students’ level of experience in solving word problems and the type of word problems used is crucial. More experienced word problem solvers have more developed cognitive schemata to solve these problems (Kintsch and Greeno, 1985). Sixth graders may be seen as experts, with a specialized knowledge base and strategies to form a representation of the problem and solve the problems top-down using their semantic schemata, whereas inexperienced word problem solvers rely more on bottom-up processing of information (De Corte et al., 1985). Typical school mathematics word problems are one-step arithmetical problems without redundant information or misleading key words. Experienced word problem solvers have developed cognitive schemata that fit such problems well, regarding structure, role, and intent of word problems (Verschaffel et al., 2000). In other words, sixth graders have probably become very skillful in selecting the appropriate cognitive scheme based on cues in the text (e.g., the word “distributed” signals the operation “division”) and insert the appropriate information from the problem statement into the empty slots (e.g., inserting 136 and 4 in the empty slots of the division operation).

Evidence for this scheme-based approach comes from studies using inconsistent word problems where the relational key words are not consistent with the required arithmetic operation (van der Schoot et al., 2009; Boonen et al., 2013). Other evidence comes from studies using “non-routine” word problems, such as “Brian and Sylvia go to the same school. Brian lives 17km away from school and Sylvia 8km. How many km apart do Brian and Sylvia live?.” These studies show that experienced students tend to answer these problems in a superficial way by selecting the most likely operation and inserting the numbers in the slots (17–8=9 in the example), without making realistic considerations such as that Brian and Sylvia could also live on different sides of the school (Verschaffel et al., 1994, 2020). In the words of Verschaffel et al. (2000, p. 13), students used “the rules of the game of word problem solving.”

To overcome this superficial problem-solving approach of “undressing” the word problem to find and execute the arithmetic operation “hidden” in the problem text, the word problems could be made less simple and straightforward. One way to make word problems more complex is by using two-step arithmetic problems that cannot be solved with one single mathematical operation, requiring students to set up and monitor a plan of solution steps (Verschaffel et al., 2020). Another way is to include irrelevant numerical information that must be ignored (Jiménez and Verschaffel, 2014; Wang et al., 2016; Leiss et al., 2019). In both ways, students cannot “skip” the mental modeling step that easily but must devote attention to analyzing the text to construct an appropriate situation model and mathematical model.

Therefore, in the current study, both one-step and two-step arithmetic word problems are included, with and without irrelevant numerical information. By including these more complex types of word problems, we aim to make the steps of constructing a situation model and transforming that into a mathematical model more salient. This should enable capturing the different problem-solving processes involved and investigate the relative influence of individual differences that have been found to impact word problem solving: reading comprehension, non-verbal reasoning, and working memory (Fuchs et al., 2015).

### Reading Comprehension

Since a key factor in constructing an adequate situation model is comprehension of the problem text, it is not surprising that reading comprehension ability and word problem solving are related (Pape, 2004; Fuchs et al., 2006, 2015; Vilenius-Tuohimaa et al., 2008; Hickendorff, 2013a,b; Leiss et al., 2019). In a detailed qualitative analysis of students’ solution processes of solving reality-based mathematics tasks, Leiss et al. (2019) found that students’ reading comprehension ability was positively related to the construction of a suitable situation model and that tasks with higher reading and situational demands impede construction of the situation model. Boonen et al. (2013) showed that the relation between reading comprehension and word problem solving was partly mediated by the skill of relational processing: the derivation of the correct relations between the solution-relevant elements from the text base of the word problem. Fuchs et al. (2015) found that word problem solving requires general language comprehension processes and word problem-specific language comprehension.

Several studies investigated whether reading comprehension is more strongly related to word problem solving than to solving symbolically presented arithmetic. In younger students (first to third graders; Fuchs et al., 2006; Hickendorff, 2013b), this stronger association was indeed found, supporting the role comprehension processes play in word problem solving. However, in sixth graders (Hickendorff, 2013a), there was no differential relation of reading comprehension with performance on the two problem types. A potential explanation is, again, the superficial, scheme-based problem-solving strategies that more experienced students use to solve these standard “dressed-up” word problems, in which they do not really strive for understanding of the problem text. In the current study, we aim to bridge the age range gap between these existing studies by using a sample of third to sixth graders, expecting to find a decrease in the extent to which reading comprehension is more strongly related to word problem solving that to symbolic arithmetic.

### Cognitive Resources

Word problems not only place demands on language abilities but also require domain-general cognitive resources. Studies with first- to third-grade students have identified several cognitive correlates of word problem solving, among which non-verbal reasoning and working memory seem the most relevant ones (Wang et al., 2016; Fuchs et al., 2020).

Non-verbal reasoning involves the ability to infer and implement rules and to identify patterns and relations (Wang et al., 2016). In word problem solving, it is relevant in targeting and organizing essential information, inferring information that is not immediately evident, and excluding irrelevant information. Wang et al. (2016) found that non-verbal reasoning is particularly important in solving word problems with irrelevant information, because the process of schema identification and application of a viable solution strategy makes strong demands on reasoning ability.

Working memory involves the ability to simultaneously store and process information (Baddeley, 1992). Recent meta-analyses showed that working memory is related to mathematics performance and that the relation with word problem solving is one of the strongest ones (Friso-Van Den Bos et al., 2013; Peng et al., 2016). In word problem solving, it plays a role in storing and manipulating multiple pieces of information in the process of constructing the situation model and transforming that into a mathematical model (Fuchs et al., 2015, 2020; Verschaffel et al., 2020).

### Current Study

Solving word problems involves multiple steps and relies on several cognitive processes. Research suggests that when students progress through primary school and thus get more experienced in solving word problems, the difference between solving standard word problems and their symbolic counterparts disappears. A potential explanation is that experienced students solve word problems in a more superficial way, relying heavily on their cognitive schemata for the semantic structures of typical school word problems. The current study aims to put this explanation to the test by seeking empirical support. To that end, we investigated the performance of students with different levels of experience (third to sixth graders) in word problems that differ in complexity (one-step vs. two-step problems; problems with and without irrelevant numerical information). By investigating the differential role that language (reading comprehension) and domain-general cognitive resources (working memory and non-verbal reasoning) play in problems in different formats and in different grades, we aim to find additional support for the differential importance of the processes.

Research question 1 addresses one-step arithmetic and focuses on the difference between problems presented symbolically or as standard word problem. We expect a performance advantage for symbolic problems over word problems in lower grades but no difference in higher grades (hypothesis 1a). Relatedly, we expect linguistic and cognitive abilities to be more strongly correlated with performance on word problems than with performance on symbolic problems in lower grades, but no differential relations in higher grades (hypothesis 1b).

Research question 2 addresses standard word problems and focuses on the difference between one-step and two-step arithmetic. We expect two-step word problems to be more difficult than one-step word problems, particularly in lower grades where students have less developed cognitive schemata available for two-step problems (hypothesis 2a). Relatedly, we expect the linguistic and cognitive individual differences to be more strongly correlated with performance on two-step problems than with performance on one-step word problems, particularly in lower grades (hypothesis 2b).

Research question 3 focuses on the difference between standard and non-standard word problems which include irrelevant numerical information. Adding irrelevant information requires cognitive resources to inhibit the irrelevant information, it requires more attention for the steps of constructing a situation model and the mathematical model, and it could lead to additional errors by erroneously using the irrelevant numerical information. Therefore, we expect non-standard word problems to be more difficult than one-step word problems, particularly in less experienced students (hypothesis 3a). Relatedly, we expect linguistic and cognitive individual differences (Wang et al., 2016) to be more strongly correlated with performance on non-standard word problems than with performance on standard word problems, particularly in lower grades (hypothesis 3b).

## Materials and Methods

### Participants

The sample consisted of 444 students (201 boys, 211 girls, 32 missing data) from seven different schools in the West of the Netherlands (30–98 students per school). There were 121 third graders, 116 fourth graders, 95 fifth graders, and 112 sixth graders. The research protocol was approved by the Institute’s IRB (number ECPW-2015 115), and only children with written parental consent participated.

As an indicator of general achievement level in mathematics and in reading comprehension, we collected the students’ most recent scores on the mathematics and reading comprehension subtests of CITO’s student monitoring system (Feenstra et al., 2010; Janssen et al., 2010; Weekers et al., 2011). This is a widely used assessment system which provides for two tests per grade (halfway and at the end of the school year). It enables schools and teachers to measure students’ achievement level and their progression. Based on nationally representative norms, students’ performance can be categorized into five quantiles: 1 (lowest 20%) through 5 (highest 20%). In the current sample, there were valid scores on the mathematics achievement subtest for 365 students, with 17.0% in category 1, 20.0% in category 2, 22.7% in category 3, 18.4% in category 4, and 21.9% in category 5. There were valid scores on the reading comprehension subtest for 362 students, with 20.7% in category 1, 19.6% in category 2, 17.7% in category 3, 20.2% in category 4, and 21.8% in category 5. These distributions did not differ by grade for either mathematics (χ2 (df=12)=15.522, *p*=0.214) or reading comprehension (χ2 (df=12)=15.025, *p*=0.240). In all, the sample is quite representative for the national population in terms of achievement level in both mathematics and reading comprehension, overall as well as per grade.

### Materials

#### Arithmetic Task

The arithmetic task consisted of 48 arithmetic problems, distributed across two booklets of 24 problems each. The problems were constructed according to two dimensions. The first dimension was *presentation format* with three types: symbolic (no text/story), standard word problems, and non-standard word problems including an irrelevant number. The second dimension was the *number of operations*: one-step problems requiring only one arithmetic operation (addition, subtraction, multiplication, or division) or two-step problems requiring two arithmetic operations (addition or subtraction combined with multiplication or division). Full crossing of these dimensions would result in six different problem types. However, two-step problems in symbolic format were not included since that would have necessitated working with brackets (e.g., (21–4)×7) which is not covered in the primary school mathematics curriculum. Table 1 presents an overview of the five problem types included in the arithmetic task.

**Table 1:** Overview of the arithmetic task.

	One-step arithmetic			Two-step arithmetic		
	Example	*k*	Alpha	Example	*k*	Alpha
Symbolic	684–248=___	16	0.889	n.a.	n.a.	n.a.
Standard word problem	In total 684 contestants from different countries participated in a cycling race. During the race 248 contestants dropped out. How many contestants reached the finish?	8	0.822	Linda worked 4days for 37 euros a day. She buys a box with DVDs for 24 euros. How much does she have left?	8	0.829
Non-standard word problem	In total 684 contestants from 10 different countries participated in a cycling race. During the race 248 contestants dropped out. How many contestants reached the finish?	8	0.812	Linda worked 4days for 37 euros a day. She buys a box with 3 DVDs for 24 euros. How much does she have left?	8	0.805

For the one-step problems, there were two problems per operation, and for each problem, there were two numerically parallel versions (e.g., version *a* 283+368; version *b* 386+238). Thus, in total, there were 4×2×2=16 problems. All 16 problems were presented in symbolic format and as word problem: either as standard word problem or as non-standard word problem. That means that students solved numerically identical problems twice, in different formats. To prevent students recalling the problems and solutions, the problems were distributed across the two different booklets, that were administered on different days. Numerically identical problems were never in the same booklet. For instance, in booklet A problem version *a* was presented in symbolic format and version *b* as standard word problem, and in booklet B, problem version *b* was presented in symbolic format and version *a* as non-standard word problem. The stories presented in the two word problems were slightly different to prevent students recognizing the story. For instance, in the one-step problem in Table 1, the cycling race was replaced by a running race. The possible combinations of word problem format (standard or non-standard), story used, and problem version *(a* or *b*) were counterbalanced across task versions.

The two-step problems involved a combination of addition or subtraction on the one hand and multiplication or addition on the other. The resulting four different combinations of operations were crossed with the two different orders (addition/subtraction first or multiplication/division first), yielding a total of eight different problems. Each problem was presented twice: as standard word problem in one booklet and as non-standard word problem in the other booklet, again with slightly different stories, for example, the DVDs were replaced by computer games in the example from Table 1 and a different name was used.

There were 16 different task versions, resulting from crossing the different counterbalancing options for the one-step problems, booklet order (booklet A first or B first), and problem order within each booklet (two pre-specified orders, one being the reverse of the other). The answers to each problem were scored as correct or incorrect. All performance scales had good reliability (Cronbach’s alpha>0.80), see Table 1.

#### Reading Comprehension

We used two different measures of reading comprehension, one based on the product of reading and the other on the process. The first measure was the earlier mentioned reading comprehension subtest of CITO’s national student monitoring system (Feenstra et al., 2010; Weekers et al., 2011). The test included various types of texts, such as informative texts and fictional texts, as well as various text genres, such as reports, letters, or poems. Students answer multiple-choice items that involve questions about the text, items where different sentences must be ordered to create a story, and fill-the-gap items where students have to select the sentence that fits best. Most questions concerned the content and meaning of the text, interleaved with questions concerning text structure. Furthermore, questions are designed to draw on three processes: comprehension, interpretation, and reflection. Reflection questions are not included before grade 4. Validity and reliability have been reported as satisfactory.

The second reading comprehension measure involved a shortened version of the Multiple-choice Online Cloze Comprehension Assessment (MOCCA; Carlson et al., 2014). This instrument is based on theories that suggest that successful reading comprehension involves the extent to which a reader can develop a coherent mental representation of a text through developing a situation model and that causal inferences are crucial (e.g., Graesser et al., 1994; van den Broek et al., 2005). The MOCCA was developed to measure comprehension processes that readers use *during* reading, thereby widening the scope of most traditional school-based reading comprehension assessments such as CITO’s test, that focus on the product rather than the process of reading comprehension. It is a paper-and-pencil multiple-choice test that consists of several short narrative texts of seven sentences. In each text, the sixth sentence is deleted, and the readers must select one of four options to complete the text. The best option requires the reader to make a causal inference that results in a coherent representation of the text. The three alternative options represent specific reading comprehension processes (i.e., paraphrases, local bridging inferences, and lateral connections).

The original MOCCA comprising 40 texts was administered to third to fifth graders (Carlson et al., 2014). Cronbach’s alpha values of selecting the correct (causal inference) option were in the 0.90s. In the current study, we used a shortened version of the MOCCA of 20 texts. Cronbach’s alpha was 0.86 in the current sample. Split by grade Cronbach’s alpha was 0.81, 0.81, 0.79, and 0.73 for grades 3 to 6, respectively.

#### Cognitive Abilities

The Raven Standard Progressive Matrices (Raven SPM, Raven et al., 1992) was used as a measure of non-verbal reasoning. The Raven SPM consists of five series of 12 diagrams or designs in which one part is missing. Students are required to select the correct part that logically completes the diagram, from six or eight options. The difficulty of the items increases when the test proceeds. Answers are scored correct (1) or incorrect (0). Internal consistency and validity have been extensively studied and found to be adequate.

The Monkey Game (Van de Weijer-Bergsma et al., 2016) was used as a measure of working memory. This is a self-reliant online computerized backward word span task. Students hear several spoken words, which they must remember and recall backward by clicking on the words presented visually in a 3×3 matrix. There are five levels of increasing difficulty determined by the number of words that must be recalled backward: two (level 1) to six (level 5). For each item, it was scored how many words were recalled in the correct backward serial position. This was transformed into a proportion correct score per item. For instance, if the item involved three words and the student recalled two words on the correct backward serial position, the proportion correct score on this item was 0.667. The reliability of the proportion correct scores in the Monkey Game was evaluated in a sample of first to sixth graders, which yielded satisfactory Cronbach’s alpha values between 0.78 and 0.85 (Van de Weijer-Bergsma et al., 2016).

### Procedure

The participating classrooms were visited by one of seven research assistants who handed out the materials and gave the instructions to the students. Per classroom there were two sessions, approximately one week apart. In session 1, the first booklet of the arithmetic task was administered as well as one or two other measures: Raven SPM, MOCCA, and/or the Monkey Game. In session 2, the second booklet of the arithmetic task was administered as well as the remaining measure(s). The arithmetic tasks, Raven SPM, and MOCCA, were administered in a classroom situation, where students worked through the tasks independently, with 35min planned for each 24-problem arithmetic task booklet, 20min for the Raven SPM, and 20min for the MOCCA. The Monkey Game was administered individually in 10min on a school laptop or computer in the classroom or in a quiet room outside the classroom.

### Analyses

To answer all research questions, multilevel logistic regression models were used with the correctness of the answer to each problem (0/1) as binary dependent variable and with a random intercept across students and across problems to account for the nesting of problems within students (for instance, see Fagginger Auer et al., 2016; Pavias et al., 2016). The analyses were run using the glmer function in the lme4-package for R (Bates et al., 2015). The individual difference measures non-verbal reasoning, working memory, and the two reading comprehension measures were sample standardized before entering the models as predictors. Predictor effects were tested using likelihood ratio tests, which involve statistically testing the improvement in model fit (log-likelihood) associated with the inclusion of a particular effect. The statistic is chi-square distributed with degrees of freedom equal to the number of parameters involved with the added effect.

## Results

**Figure 1:**
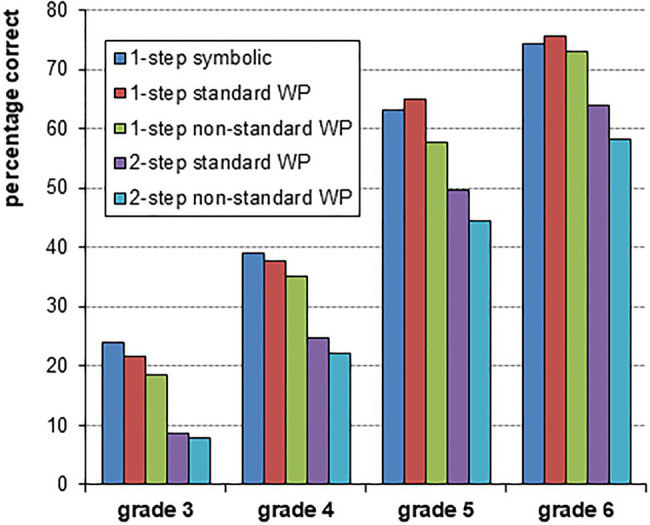
Performance means on the five problem types by grade.

Descriptive statistics of the measures are presented in Table 2, and the results for the arithmetic tasks are also presented graphically in Figure 1. On all measures, there were significant differences between grades (*p*s<0.001). For CITO’s reading comprehension, differences between grades could not be tested because it involved grade-specific norm-referenced scores. Table 3 presents the correlations between the measures (except CITO’s reading comprehension). All measures were significantly correlated (*p*s<0.001). The two different reading comprehension measures MOCCA and CITO correlated 0.492 in grade 3; 0.507 in grade 4, 0.384 in grade 5; and 0.409 in grade 6 (*p*s<0.001).

**Table 2:** Descriptive statistics of the measures: Means and SD’s (between brackets).

	Grade 3	Grade 4	Grade 5	Grade 6	Total
One-step symbolic^a^	24.0	39.0	63.3	74.3	49.0
	(19.35)	(21.09)	(24.33)	(18.77)	(28.86)
One-step standard WP^a^	21.6	37.8	65.1	75.6	48.8
	(19.90)	(24.36)	(25.78)	(20.09)	(31.23)
One-step non-standard WP^a^	18.5	35.1	57.6	73.1	45.0
	(20.29)	(23.01)	(27.85)	(20.84)	(31.20)
Two-step standard WP^a^	8.8	24.7	49.6	64.1	35.6
	(12.67)	(22.14)	(29.14)	(27.06)	(31.77)
Two-step non-standard WP^a^	8.0	22.1	44.6	58.1	32.2
	(13.50)	(20.68)	(29.61)	(25.26)	(29.95)
Working memory^b^	0.463	0.522	0.576	0.606	0.538
	(0.1470)	(0.1293)	(0.1187)	(0.1205)	(0.1409)
Non-verbal reasoning^c^	34.6	36.5	41.4	42.0	38.4
	(6.60)	(7.23)	(5.50)	(5.83)	(7.10)
Reading comprehension (MOCCA)^d^	7.8	11.4	13.4	15.3	11.8
	(4.37)	(4.30)	(3.88)	(3.20)	(4.87)
Reading comprehension (CITO)^e^	3.20	2.91	2.91	3.10	3.03
	(1.446)	(1.508)	(1.379)	(1.452)	(1.451)

a Percentage correct on the different problem types in the arithmetic task (0–100).

b Mean proportion correct on the backward recall items of the Monkey Game (0–1).

c Number of items correct in the Raven SPM (0–60).

d Number correct on the MOCCA (0–20).

e Grade-specific norm score CITO reading comprehension test (1=lowest quintile; 5=highest quintile).

**Table 3:** Correlations between the measures.

	1	2	3	4	5	6	7
1. One-step symbolic							
2. One-step standard WP	0.867						
3. One-step non-standard WP	0.838	0.867					
4. Two-step standard WP	0.822	0.811	0.822				
5. Two-step non-standard WP	0.785	0.784	0.788	0.848			
6. Working memory	0.460	0.452	0.455	0.470	0.456		
7. Non-verbal reasoning	0.549	0.543	0.528	0.526	0.509	0.467	
8. Reading comprehension (MOCCA)	0.573	0.557	0.570	0.609	0.555	0.393	0.447

All *p*s<0.001.

### Standard Word Problems Versus Symbolic Problem

Research question 1 involves the comparison of standard, one-step word problems with their symbolically presented counterparts. Table 4 shows the model-building steps of the multilevel logistic regression models. To test hypothesis 1a, students’ grade (3, 4, 5, or 6) and problem format (word problem vs. symbolic format) were added as predictors to an empty model with only random intercepts across students and across problems. The main effect of grade was significant (all pairwise differences were significant), whereas the main effect of problem format was not. The interaction effect between grade and problem format was significant (*p*=0.043). *Post hoc* comparisons revealed that there was a non-significant performance advantage of symbolic problems in grade 3 (*β*=−0.26, *z*=−0.49) and in grade 4 (*β*=−0.11, *z*=−0.21) which turned into a non-significant performance advantage of word problems in grade 5 (*β*=0.15, *z*=0.28) and in grade 6 (*β*=0.14, *z*=0.28), see also Figure 1. This partly confirms hypothesis 1a.

**Table 4:** Statistical tests for research question 1: standard word problems versus symbolic problems.

	Fixed effects	LL	#p	LR	*χ* ^2^	*df*	*p*
**Hypothesis 1a**							
M0	-	−5170.9	3				
M1	Grade	−5024.5	6	M1-M0	292.824	3	<0.001
M2	Grade+Pr.Format	−5024.5	7	M2-M1	0.004	1	0.95
M3	Grade*Pr.Format	−5020.4	10	M3-M2	8.146	3	0.043
**Hypothesis 1b: reading comprehension process (MOCCA)**							
M4a	Grade+Pr.Format	−5000.3	7				
M5a	Grade+Pr.Format+MOCCA	−4980.2	8	M5a-M4a	46.144	1	<0.001
M6a	Grade+Pr.Format*MOCCA	−4979.0	9	M6a-M5a	2.312	1	0.13
M7a	Grade*Pr.Format*MOCCA	−4978.0	15	M7a-M6a	2.018	6	0.92
**Hypothesis 1b: reading comprehension product (CITO)**							
M4b	Grade+Pr.Format	−4162.5	7				
M5b	Grade+Pr.Format+CITO	−4140.4	8	M5b-M4b	44.130	1	<0.001
M6b	Grade+Pr.Format*CITO	−4140.4	9	M6b-M5b	0.818	1	0.37
M7b	Grade*Pr.Format*CITO	−4138.6	15	M7b-M6b	2.71	6	0.82
**Hypothesis 1b: non-verbal reasoning (RAVEN)**							
M4c	Grade+Pr.Format	−5024.5	7				
M5c	Grade+Pr.Format+RAVEN	−4981.8	8	M5c-M4c	85.318	1	<0.001
M6c	Grade+Pr.Format*RAVEN	−4979.8	9	M6c-M5c	3.990	1	0.046
M7c	Grade*Pr.Format*RAVEN	−4976.1	15	M7c-M6c	7.424	6	0.28
**Hypothesis 1b: working memory (WM)**							
M4d	Grade+Pr.Format	−4899.9	7				
M5d	Grade+Pr.Format+WM	−4875.4	8	M5d-M4d	48.930	1	<0.001
M6d	Grade+Pr.Format*WM	−4874.4	9	M6d-M5d	2.154	1	0.14
M7d	Grade*Pr.Format*WM	−4871.3	15	M7d-M6d	6.056	6	0.42

LL, log-likelihood; #p, number of parameters; LR, likelihood ratio test; *χ*^2^, test statistic LR-test; Pr.Format, problem format (word problem or symbolic problem).

To address hypothesis 1b, we tested each individual difference measure in a separate run of analyses, starting with adding the main effect of that measure (M5), then testing whether there was a differential effect according to problem format (M6), and finally testing whether this differential effect according to problem format depended on students’ grade (M7). Both reading comprehension measures and both cognitive abilities were significantly associated with mathematics performance, but only non-verbal reasoning was differentially related to word problem solving versus symbolic problems. As expected, the association with word problem solving was significantly stronger than the association with solving symbolic problems: *β*_WP_=0.77, *z*=8.60 vs. *β*_symb_=0.65, *z*=9.15; *z*_difference_=2.02. This differential relation did not depend on grade, however. Hypothesis 1b was therefore only partly accepted: Non-verbal reasoning was stronger related to word problem solving than to solving symbolic problems across all grades but reading comprehension and working memory were not related differentially to performance on the two types of problems.

### Two-Step Versus One-Step Arithmetic Word Problems

Research question 2 involves the comparison of one-step and two-step arithmetic word problems. Table 5 shows the model-building steps of the multilevel logistic regression models. To test hypothesis 2a, students’ grade (3, 4, 5, or 6) and number of arithmetic steps (one step vs. two steps) were added as predictors to an empty model with only random intercepts. The main effect of grade was significant, whereas the main effect of arithmetic steps and the interaction effect between grade and arithmetic steps were not. Hypothesis 2a was therefore rejected: Two-step word problems were not more difficult than one-step word problems.

**Table 5:** Statistical tests for research question 2: two-step versus one-step word problems.

	Fixed effects	LL	#p	LR	χ^2^	*df*	*p*
**Hypothesis 2a**							
M0	-	−3465.9	3				
M1	Grade	−3309.0	6	M1-M0	313.778	3	<0.001
M2	Grade+Steps	−3307.5	7	M2-M1	3.044	1	0.081
M3	Grade*Steps	−3305.5	10	M3-M2	4.014	3	0.26
**Hypothesis 2b: reading comprehension process (MOCCA)**							
M4a	Grade+Steps	−3295.1	7				
M5a	Grade+Steps+MOCCA	−3261.6	8	M5a-M4a	63.328	1	<0.001
M6a	Grade+Steps*MOCCA	−3255.8	9	M6a-M5a	6.480	1	0.011
M7a	Grade*Steps*MOCCA	−3254.8	15	M7a-M6a	0.892	6	0.99
**Hypothesis 2b: reading comprehension product (CITO)**							
M4b	Grade+Steps	−2746.9	7				
M5b	Grade+Steps+CITO	−2715.2	8	M5b-M4b	88.702	1	<0.001
M6b	Grade+Steps*CITO	−2712.0	9	M6b-M5b	0.156	1	0.69
M7b	Grade*Steps*CITO	−2711.5	15	M7b-M6b	6.686	6	0.35
**Hypothesis 2b: non-verbal reasoning (RAVEN)**							
M4c	Grade+Steps	−3307.5	7				
M5c	Grade+Steps+RAVEN	−3263.1	8	M5c-M4c	58.582	1	<0.001
M6c	Grade+Steps*RAVEN	−3263.0	9	M6c-M5c	1.856	1	0.17
M7c	Grade*Steps*RAVEN	−3259.7	15	M7c-M6c	2.810	6	0.83
**Hypothesis 2b: working memory (WM)**							
M4d	Grade+Steps	−3227.5	7				
M5d	Grade+Steps+WM	−3198.2	8	M5d-M4d	66.958	1	<0.001
M6d	Grade+Steps*WM	−3197.3	9	M6d-M5d	11.604	1	0.001
M7d	Grade*Steps*WM	−3195.9	15	M7d-M6d	2.032	6	0.92

LL, log-likelihood; #p, number of parameters; LR, likelihood ratio test; *χ*^2^, test statistic LR-test; Steps, number of arithmetic steps (one or two).

To address hypothesis 2b, we again tested each individual difference measure in a separate run of analyses. Both reading comprehension measures and both cognitive abilities were significantly associated with mathematics performance, but the two reading comprehension measures were differentially related to word problem solving versus symbolic problems. As expected, the association with two-step arithmetic word problems was significantly stronger than the association with one-step arithmetic word problems for the CITO measure (*β*_2step_=0.71, *z*=8.48 vs. *β*_1step_=0.52, *z*=6.35; *z*_difference_=2.58, *p*=0.010) as well as for the MOCCA measure (*β*_2step_=0.83 and *β*_1step_=0.57; *z*=3.45, *p*<0.001). This differential relation did not depend on grade, however. Hypothesis 2b was therefore only partly accepted: Reading comprehension was stronger related to solving two-step word problems than to solving one-step word problems across all grades but working memory and non-verbal reasoning were not related differentially to performance on the two types of problems, across all grades.

### Non-standard Versus Standard Word Problems

Research question 3 involves the comparison of standard word problems with non-standard word problems that include irrelevant numerical information. Table 6 shows the model-building steps of the multilevel logistic regression models. To test hypothesis 3a, students’ grade (3, 4, 5, or 6), number of arithmetic steps (one step vs. two steps) and problem type (standard vs. non-standard word problems) were added as predictors to an empty model with only random intercepts. The main effect of grade was significant. In this model, the main effect of arithmetic steps was significant (*β*=−0.92, z=−2.73, *p*=0.006) with lower performance on two-step problems than on one-step problems. The main effect of problem type was not significant nor was the interaction between students’ grade and problem type. Hypothesis 3a was therefore rejected: Non-standard word problems with irrelevant numerical information were not more difficult than standard word problems.

**Table 6:** Statistical tests for research question 3: non-standard versus standard word problems.

	Fixed effects	LL	#p	LR	χ^2^	*df*	*p*
**Hypothesis 3a**							
M0	-	−6617.3	3				
M1	Grade	−6460.0	6	M1-M0	314.624	3	<0.001
M2	Steps+Grade	−6456.7	7	M2-M1	6.606	1	0.010
M3	Steps+Grade+Pr.Type	−6456.4	8	M3-M2	0.640	1	0.42
M5	Steps+Grade*Pr.Type	−6453.7	11	M4-M3	5.372	3	0.15
**Hypothesis 3b: reading comprehension process (MOCCA)**							
M5a	Steps+Grade+Pr.Type	−6431.2	8				
M6a	Steps+Grade+Pr.Type+MOCCA	−6396.8	9	M6a-M5a	68.718	1	< 0.001
M7a	Steps+Grade+Pr.Type*MOCCA	−6395.7	10	M7a-M6a	2.282	1	0.13
M8a	Steps+Grade*Pr.Type*MOCCA	−6394.7	16	M8a-M7a	2.026	6	0.92
**Hypothesis 3b: reading comprehension product (CITO)**							
M5a	Steps+Grade+Pr.Type	−5364.8	8				
M6a	Steps+Grade+Pr.Type+CITO	−5330.0	9	M6b-M5b	69.726	1	< 0.001
M7a	Steps+Grade+Pr.Type*CITO	−5329.9	10	M7b-M6b	0.056	1	0.81
M8a	Steps+Grade*Pr.Type*CITO	−5327.1	16	M8b-M7b	5.720	6	0.46
**Hypothesis 3b: non-verbal reasoning (RAVEN)**							
M5a	Steps+Grade+Pr.Type	−6456.4	8				
M6a	Steps+Grade+Pr.Type+RAVEN	−6407.7	9	M6cb-M5c	97.412	1	<0.001
M7a	Steps+Grade+Pr.Type*RAVEN	−6406.8	10	M7c-M6c	1.720	1	0.19
M8a	Steps+Grade*Pr.Type*RAVEN	−6403.9	16	M8c-M7c	5.858	6	0.44
**Hypothesis 3b: working memory (WM)**							
M4d	Steps+Grade+Pr.Type	−6303.3	8				
M5d	Steps+Grade+Pr.Type+WM	−6270.2	9	M6d-M5d	66.314	1	<0.001
M6d	Steps+Grade+Pr.Type*WM	−6269.9	10	M7d-M6d	0.486	1	0.49
M7d	Steps+Grade*Pr.Type*WM	−6266.8	16	M8d-M7d	6.202	6	0.40

LL, log-likelihood; #p, number of parameters; LR, likelihood ratio test; *χ*^2^, test statistic LR-test; Steps, number of arithmetic steps (one or two); Pr.Type, problem type (standard or non-standard word problem).

To address hypothesis 3b, we again tested each individual difference measure in a separate run of analyses. Both reading comprehension measures and both cognitive abilities were significantly associated with mathematics performance, but not differentially with the two word problem types. Hypothesis 3b was therefore rejected: There were no differential relations with the individual difference measures with performance on standard versus non-standard word problems, across all grades.

## Discussion

Arithmetic word problems require multiple processes, of which constructing a situation model of the problem text and translating that into a mathematical model are the most salient ones. Therefore, word problems are more difficult to solve and make additional linguistic and cognitive demands compared to arithmetic problems in symbolic format, as studies in first to third graders show (Fuchs et al., 2006; Hickendorff, 2013b; Wang et al., 2016). However, research suggests that as students progress through primary school and get more experienced in solving word problems, these extra steps may have less impact on their performance and solution strategies, which could possibly be explained by a heavier reliance on their cognitive schemata for typical one-step arithmetic word problems (Hickendorff, 2013a). The current study addressed this hypothesis by extending the age range, making word problems more complex, and including a more varied set of individual differences, tapping into reading comprehension and domain-general cognitive resources.

The first research question involved the comparison of standard, one-step arithmetic word problems with their counterparts in symbolic format. Findings showed that although performance increased across grades, within each grade these two problem formats were just as difficult. However, the non-significant performance advantage of symbolic problems in grades 3–4 flipped into a non-significant performance advantage of standard word problems in grades 5–6. This significant decrease in the performance advantage of symbolic problems is consistent with our expectations that the steps of constructing a situation model and translating that into a mathematical model, which are expected to make word problems relatively difficult, are less prominent when students get more experienced in word problem solving. From the four individual difference measures, only non-verbal reasoning showed a stronger association with word problem solving than with solving problems in symbolic format, which is consistent with the expectations. The expectation that this depended on grade was not supported. Furthermore, working memory and the two reading comprehension measures were not differentially related to performance on the two problem formats, although we did expect a stronger relation with word problem solving. All in all, there seem to be very little differences between standard word problems and their counterparts in symbolic format in performance as well as in their demands on cognitive and language resources, across all grades. This implies that already in third-grade students seem helped nor hampered by the realistic stories presented in the word problems when it concerns standard one-step arithmetic word problems, replicating the findings of Hickendorff (2013a) and extending that to younger students.

Another manipulation was to make the word problems more complex to diminish the possibilities that they can be solved with superficial strategy of “undressing” the word problem to find the “hidden” arithmetic problem without striving for understanding of the problem situation in the text (Leiss et al., 2019; Verschaffel et al., 2020). Problems were made more complex in two ways: by requiring two-step arithmetic (research question 2) and by including irrelevant numerical information (research question 3). Contrary to our expectations, neither of the two manipulations made the problems more difficult. However, two-step word problems were more strongly related to the two reading comprehension measures than one-step word problems, whereas there were no differential relations with working memory and non-verbal reasoning. This suggests that comprehension processes are more relevant than domain-general cognitive processes in setting up and monitoring a plan of solution steps in solving two-step word problems. Since this held across grades, there was no support for the hypothesis that the language demands lessen when students get more experienced.

The non-standard word problems with irrelevant numerical information did not make additional demands on language or domain-general resources, contrary to our expectations but for language and working memory consistent with findings in second graders (Wang et al., 2016). This implies that students were not hindered by the extra numerical information that they had to ignore. In the Netherlands, students probably encounter a wide variety of realistic situations, because Realistic Mathematics Education (RME) is the dominant instructional approach. In RME, realistic situations play a large role throughout the instructional trajectory, and mathematizating reality is an important goal (Gravemeijer and Doorman, 1999; Van den Heuvel et al., 2014). Consequently, Dutch students may have encountered a wider variety of word problems than students from countries with other instructional approaches. Further studies could investigate how Dutch students solve other types of non-standard word problems such as the non-routine problems from Verschaffel et al. (1994) or problems with more than one piece of irrelevant information.

### Educational Implications

The current findings have several implications for theory and instruction. For theoretical models of word problem solving, it is important to take the level of experience of the problem solver into account. The current study suggests that the steps of constructing a situation model and translating that into a mathematical model are less salient for older students with more experience in word problem solving than studies with younger students indicate. A related implication is that an instructional approach in which students are taught to map a novel problem to one of their problem schemata may run the risk of students looking for the “hidden” problem without striving for true understanding of the problem situation. An important question is then to what extent one can then truly speak of mathematizing reality, which is one of the cornerstones of mathematics education reform such as RME.

Another implication involves the role of comprehension processes, which seem to be more important in two-step arithmetic word problems than in one-step arithmetic word problems but had no differential impact on non-standard versus standard word problems. If researchers or teachers want to impact comprehension processes in word problem solving, we recommend using multiple-step arithmetic problems to make the standard, one-step word problems more challenging. A final point of discussion is that word problems and assessments including many word problems are sometimes criticized for making heavy demands on students’ language abilities, thereby disadvantaging students with lower language skills. However, the current study suggests that this does not hold for one-step arithmetic word problems, probably because the linguistic demands of such word problems are not that challenging for upper grade primary students.

### Limitations

Although there are several strong points of the study’s methodology, including the large sample size and the careful matching of characteristics of the different problem types, there are of course also limitations. A first set of limitations related to the problems. Since it was not possible to include two-step arithmetic word problems in symbolic format because students did not encounter such problems in their mathematics instruction, we could not compare the processes involved in two-step word problems with those of two-step arithmetic in symbolic format. This study could be replicated in students at the beginning of secondary education where they did learn how to solve such problems, addressing the question whether two-step word problems are more difficult than two-step arithmetic problems in symbolic format. A further limitation was that the linguistic complexity of the problems was not monitored whereas this has effects on the linguistic demands of the problems (Abedi and Lord, 2001).

A second set of limitations concerns the measures. Other studies have chosen different tests for the same constructs (Fuchs et al., 2015; Wang et al., 2016) which could lead to slightly different results. Furthermore, there are also other cognitive correlates of word problem solving that were not included in the current study, such as processing speed (Wang et al., 2016) and inhibitory control, which is increasingly considered to be important in mathematics learning in general and in word problem solving in particular (Van Dooren and Inglis, 2015), and for which it would be particularly interesting to assess its impact influence on problems with irrelevant information that has to be ignored.

A final limitation is that there is no information on the solution strategies students used, since only the answer was scored and analyzed. Consequently, there is no direct test of the suggested mechanism that the steps of constructing a situation model and translating that into a mathematical model are less salient in upper grade students than previous studies reported in younger students. It is therefore not possible to rule out other explanations, such as increased conceptual knowledge in older students aiding constructing the mathematical model. Future research could implement a smaller-scale qualitative study in which students solve the different problem types by thinking aloud. Such process data could give more insights into the steps taken in constructing a situational and a mathematical model and could also yield implications for the improvement of instruction.

### Conclusion

Limitations aside, the current study’s findings are consistent with the hypothesis that the steps of constructing a situation model and translating that into a mathematical model, and the demands on language comprehension and domain-general cognitive resources involved with those steps, are less salient in upper grade students than previous studies reported in younger students. Third- to sixth-grade students seem helped nor hindered by situating the arithmetic problem in a story, even if that story includes irrelevant numerical information. Comprehension processes seem particularly relevant in two-step arithmetic word problems.

## Data Availability Statement

The data supporting the conclusions of this paper are uploaded in the DataVerseNL repository: https://doi.org/10.34894/7KI4M9. Requests for further information should be addressed to Marian Hickendorff, hickendorff@fsw.leidenuniv.nl.

## Ethics Statement

The studies involving human participants were reviewed and approved by the Leiden University Institute of Education and Child Studies Commissie Ethiek. Written informed consent to participate in this study was provided by the participants’ legal guardian/next of kin.

## Author Contributions

MH: conceptualization, methodology, formal analysis, writing – original draft, and writing – review and editing.

## Conflict of Interest

The author declares that the research was conducted in the absence of any commercial or financial relationships that could be construed as a potential conflict of interest.

## Publisher’s Note

All claims expressed in this article are solely those of the authors and do not necessarily represent those of their affiliated organizations, or those of the publisher, the editors and the reviewers. Any product that may be evaluated in this article, or claim that may be made by its manufacturer, is not guaranteed or endorsed by the publisher.
